# PARP Inhibitors in the Treatment of Prostate Cancer: An Analysis of the Clinical Trial Landscape

**DOI:** 10.1002/cam4.71298

**Published:** 2025-10-30

**Authors:** Yanru Chen, Junjie Cao, Rong Huang, Junyi Lou, Junxian Gu, Zining Luo, Tianliang Yao, Jiebin Xie

**Affiliations:** ^1^ Affiliated Hospital of North Sichuan Medical College Nanchong Sichuan China; ^2^ North Sichuan Medical College Nanchong Sichuan China; ^3^ Department of Electronic Engineering Faculty of Engineering, The Chinese University of Hong Kong Hong Kong SAR China

**Keywords:** clinical landscape analysis, metastatic castration‐resistant prostate cancer (mCRPC), PARP inhibitors, prostate cancer, targeted therapy

## Abstract

**Background:**

Prostate cancer remains one of the most prevalent cancers among men worldwide, particularly in the context of metastatic castration‐resistant prostate cancer (mCRPC), which poses significant treatment challenges. PARP inhibitors offer a promising therapeutic option for patients with homologous recombination repair (HRR) deficiencies.

**Methods:**

This study systematically analyzed 630 registered clinical trials related to prostate cancer and PARP inhibitors as of April 25, 2025. A total of 109 trials were included, focusing on key information such as year of initiation, trial phase, targeted populations, and study designs.

**Results:**

Our findings indicate a significant increase in clinical trials involving PARP inhibitors from 2012 to 2025. Multi‐national collaborative studies accounted for 39.4% of the trials, with the United States being the principal contributing country. The majority of trials are concentrated on targeting PARP1 and PARP2 at various phases of development.

**Conclusions:**

PARP inhibitors have demonstrated breakthrough advancements in the treatment of mCRPC; however, challenges such as resistance and the need for personalized therapies persist. Future research should emphasize target identification and the exploration of combination therapy strategies to enhance clinical efficacy.

## 1 Introduction

Prostate cancer, a type of malignant tumor that severely threatens human health, has become a major challenge in global public health [1]. According to statistics, prostate cancer is the most common nonskin cancer among men in the United States. Globally, prostate cancer is the second most common cancer in men, with approximately 1.5 million new cases diagnosed annually. With population aging and growth, the number of cases is expected to rise to approximately 2.4 million by 2040, with deaths reaching 712 000 [2]. The clinical presentation of prostate cancer patients varies. Approximately 75% of patients are diagnosed with localized prostate cancer, for which the 5‐year survival rate is nearly 100%. However, approximately 10% of patients are diagnosed with metastatic prostate cancer at the time of diagnosis, with a 5‐year survival rate of only 37% [3].

Currently, the main therapeutic drugs for prostate cancer include the chemotherapy agent docetaxel, which has been widely used since 2004, as well as subsequent androgen receptor signaling inhibitors such as enzalutamide and abiraterone acetate [4, 5]. Although the aforementioned therapeutic drugs provide survival benefits, prostate cancer remains lethal during the metastatic castration‐resistant prostate cancer (mCRPC) phase [6].

With a deeper understanding of the molecular mechanisms of the disease and the discovery of different subtypes of prostate cancer, poly (ADP‐ribose) polymerase (PARP) inhibitors, which have emerged in recent years, offer new hope. The primary mechanism of action of these drugs is to selectively induce tumor cell death through a synthetic lethality effect in cancer cells carrying homologous recombination repair (HRR) defects, such as those with BRCA1/2 mutations [7]. It is noteworthy that approximately one‐quarter of metastatic castration‐resistant prostate cancer (mCRPC) patients have cancer cells with HRR pathway defects, making PARP inhibitors a highly promising targeted treatment option for this group [8, 9]. With the maintenance of good efficacy and manageable safety, PARP inhibitors are expected to expand the patient population benefiting from treatment through combination therapy strategies, showing broad prospects for clinical application [9].

Clinical trials for PARP inhibitors in the treatment of prostate cancer are rapidly increasing worldwide. Although some clinical results and mechanistic discussions on single‐drug therapies have emerged in the past, there is still a lack of comprehensive evidence to systematically review the global research and development landscape. Therefore, this study innovatively integrates data from global clinical trial registration platforms to provide a multidimensional descriptive analysis of the overall layout, phase distribution, and drug development status of current trials, offering references for the macro‐level optimization of future clinical trial designs.

## 2 Methods

**FIGURE 1:**
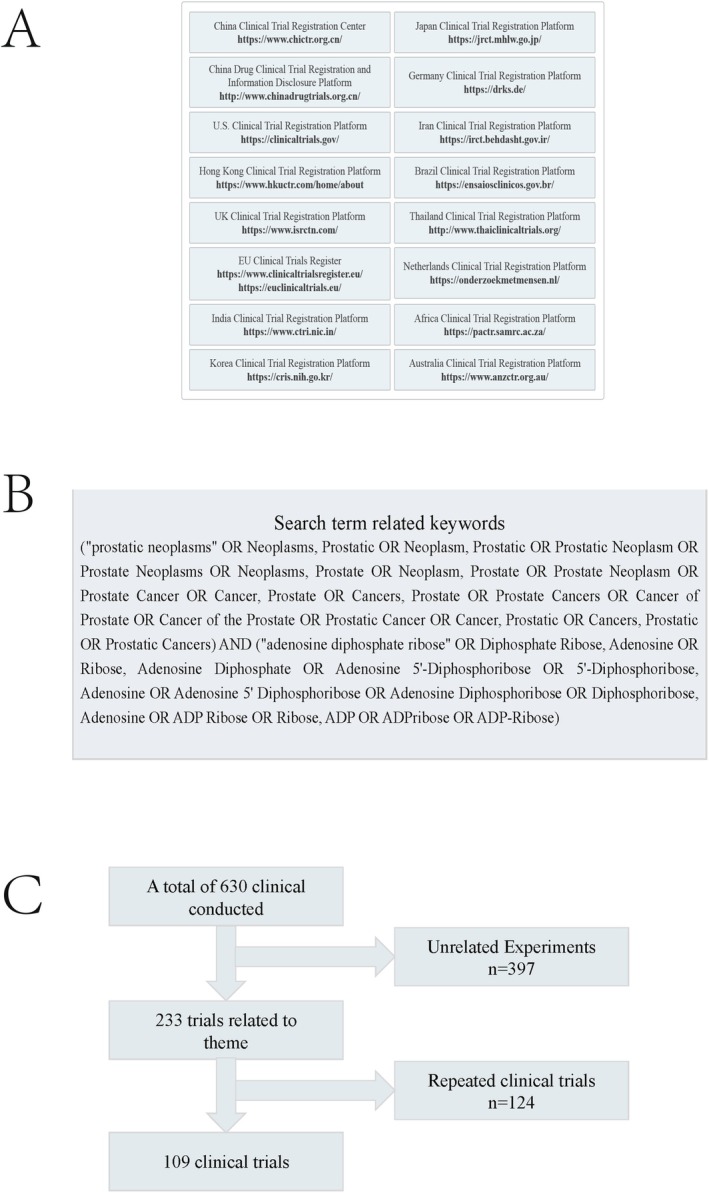
Clinical trial sources and inclusion and exclusion flowchart. (A) Clinical trial sources; (B) search term related keywords; (C) inclusion and exclusion flowchart.

This study systematically analyzed clinical trials of the use of PARP inhibitors in the treatment of prostate cancer by searching 16 clinical trial registries (Figure 1A). A comprehensive screening of clinical trials registered as of April 25, 2025, was conducted using “Prostatic Neoplasms” and “Poly(ADP‐ribose) Polymerase Inhibitors” as search keywords (Figure 1B). A total of 630 potential studies were initially identified. Trials unrelated to clinical research or those with duplicate registrations were excluded, and only clinical studies involving PARP inhibitors as a monotherapy or in combination with other regimens for the treatment of prostate cancer were included (Figure 1C). Data were extracted on 16 dimensions, including the start year, trial phase, target mechanisms, study design type, research progress status, primary endpoint characteristics, drug names, and trial regions, among others. Missing dimensions in the trial registry were completed by accessing the original trial registration information or contacting the potential registrants. For statistical analysis, descriptive statistics were primarily used, and all statistical analyses were performed using SPSS 26.0. Frequency counts of categorical variables were conducted, with results presented as numbers (percentages). Data extraction was performed by two researchers (J. Cao and Y. Chen), and any discrepancies or disputes were resolved through discussions organized by a third researcher (R. Huang), who made the final decision.

## 3 Results

After excluding duplicate records and trials unrelated to the topic, we ultimately identified 109 trials that met the inclusion criteria (Figure 1). A temporal analysis revealed that all trials were conducted between 2012 and 2025, with the highest number of trials occurring in 2019 (*n* = 24), averaging approximately 9 trials per year.

### 3.1 International Collaboration and Population Characteristics

**FIGURE 2:**
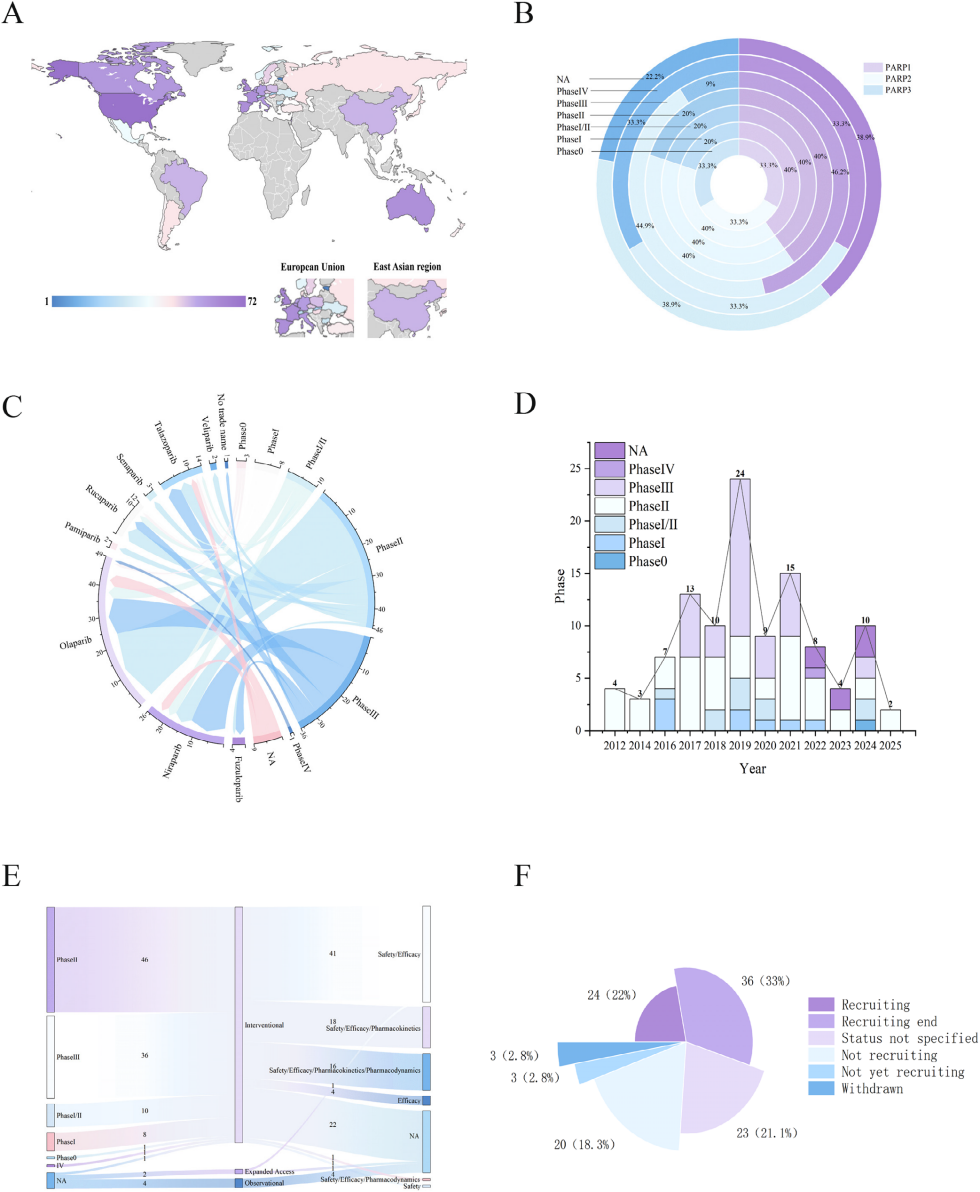
Comprehensive analysis of clinical trials of poly(ADP–ribose) polymerase inhibitors for the treatment of prostatic neoplasms. (A) Distribution of PARP inhibitors in the treatment of prostate cancer by country/region; (B) distribution of PARP inhibitor targets by clinical stage; (C) interaction and data flow between different clinical trial stages and PARP inhibitors; (D) distribution of PARP inhibitor clinical trial registration dates by clinical stage; (E) data flow and correlation strength between clinical trial stages, trial types, and endpoint types in current clinical trials of PARP inhibitors; (F) distribution of the current clinical trial status of PARP inhibitors in the treatment of prostate cancer.

Clinical trials of the use of PARP inhibitors for the treatment of prostate cancer have demonstrated a significant trend toward international collaboration (Figure 2A). Multinational, multicenter trials (*n* = 43) represented 39.4%, with the largest and most diverse study (NCT04497844) spanning 33 countries across Asia, Europe, North America, South America, Africa, and Oceania. This study enrolled 696 participants from diverse ethnic groups, including East Asian, Caucasian, African, and mixed populations, reflecting careful consideration of regional population differences. From a national perspective, the United States leads in this field, conducting 34 independent trials and participating in 38 collaborative trials, totaling 72 studies. The United Kingdom, France, Spain, and Australia follow closely, conducting between 36 and 40 related trials. Thus, clinical research on PARP inhibitors for prostate cancer has formed a research landscape led by Western countries, with active participation from emerging economies, advancing through global multicenter collaboration.

### 3.2 Target Mechanisms and Stage Characteristics

In terms of molecular targets, most drugs act on both PARP1 and PARP2, whereas PARP3 is targeted less frequently (Figure 2B). In the early phases (Phase 0) and later phases (Phase IV), there is only one study involving three targets: PARP1, PARP2, and PARP3. In early‐phase studies (Phase I, Phase I/II), PARP1 and PARP2 are the primary targets. In mid‐phase studies (Phase II and Phase III), PARP1 and PARP2 dominate, but the overall number of trials increases. In the not applicable (NA) stage, the number of studies targeting all three PARP targets decreases, but PARP1 and PARP2 remain the primary targets.

### 3.3 Drug Names and Stage Characteristics

The PARP inhibitors used in the trials included fuzuloparib, niraparib, olaparib, pamiparib, rucaparib, senaparib, talazoparib, and veliparib (Figure 2C). Olaparib is involved across all stages, with the most trials in Phase II (*n* = 24), followed by Phase III (*n* = 12), Phase I/II (*n* = 4), and Phase I (*n* = 3), with Phase 0 and Phase IV having the fewest (*n* = 1). Niraparib has the most trials in Phase III (*n* = 12), followed by Phase II (*n* = 6) and Phase I/II (*n* = 3), with Phase I having the fewest (*n* = 2). Talazoparib and rucaparib are involved in all phases except Phase IV, whereas fuzuloparib, senaparib, pamiparib, and veliparib are involved mainly in Phase II and Phase III, with relatively few trials. Overall, Phase II includes the largest number of drugs (*n* = 46), underscoring its role as a key focus of research. Additionally, multiple drugs are present across all stages, which may suggest extensive development of combination therapies or collaborative research for prostate cancer. In short, the chord diagram illustrates the allocation of resources, evolving research priorities, and market competition trends across different phases of drug development.

### 3.4 Trial Phases and Temporal Characteristics

In terms of trial timing, relevant trials were conducted as early as 2012 (Figure 2D). Early studies were predominantly in Phase II, including those from 2012 (*n* = 4) and 2014 (*n* = 3). In 2016, most studies were conducted in Phases I and II (*n* = 7). In 2017, the number of Phase II studies increased (*n* = 7), and Phase III studies began to appear (*n* = 6). In 2018, studies included Phase I/II (*n* = 2), Phase II (*n* = 5), and Phase III (*n* = 3) trials. In 2019, the number of studies peaked (*n* = 24), with Phase III studies being the most common (*n* = 15). From 2020 to 2023, except for a slight increase in 2021 (*n* = 15), the number of studies generally declined, with a focus on Phases II and III. In 2024, the number of studies rose again (*n* = 10), with a relatively balanced distribution across phases. Currently, in 2025, only Phase II studies are ongoing (*n* = 2). Overall, the number of Phase III trials peaked in 2019 and gradually decreased, whereas the number of Phase II trials remained variable, with some studies classified as NA (not applicable) and increasing diversity in the stages of research.

### 3.5 Trial Types, Phases, and Endpoint Characteristics

Interventional trials play a central role across all clinical development phases, particularly in Phase II (*n* = 46) and Phase III (*n* = 36), highlighting the key role of active intervention designs in the drug development process (Figure 2E). Safety and efficacy evaluation remain a core focus across all phases and is always a primary research concern. Notably, as development progressed, the emphasis on pharmacokinetic and pharmacodynamic parameters increased, reflecting increasingly refined and in‐depth study designs. Additionally, the presence of observational trials (*n* = 4) and compassionate use trials (*n* = 2) indicates that postmarket surveillance and real‐world evidence studies have gained importance in this field.

### 3.6 Trial Status Characteristics

Regarding trial progress, 33% of the trials were in the “complete recruitment” status, indicating that most studies had finished participant enrollment and were entering the data processing or results summarization stages (Figure 2F). Twenty‐three percent of the trials had an unclear current progression status. Twenty‐two percent of the trials are in the recruitment phase, indicating that relevant studies are still ongoing. Trials in the “not recruiting,” “not yet recruiting,” and “withdrawn” statuses represent 20%, 3%, and 3%, respectively, indicating that some studies have terminated recruitment, have not yet started, or have been withdrawn for specific reasons.

### 3.7 Treatment Efficacy and Safety

Among PARP inhibitors approved for the treatment of mCRPC, based on preliminary analyses of registration trials including NCT02987543 (Olaparib), NCT02952534 (Rucaparib), NCT02854436 (Niraparib), and NCT03395197 (Talazoparib), the efficacy of PARP inhibitors shows marked drug‐specific differences (Table 1). In cohort A with BRCA1, BRCA2, or ATM alterations, Olaparib achieved a median overall survival of 18.5 months, superior to 15.1 months in the control group (hazard ratio for death, 0.64; *p* = 0.02); in the overall population (cohort A and cohort B with other specified gene alterations), the median overall survival was 7.4 months versus 3.9 months in the control group. In patients with BRCA mutations, Rucaparib showed overall better efficacy, with an objective response rate of 45.7% and a median progression‐free survival of 10.7 months. Niraparib demonstrated better therapeutic effect in patients harboring BRCA1/2 mutations (progression‐free survival 8.08 months in the mutation group vs. 3.73 months in the non‐mutated group). Talazoparib combined with enzalutamide achieved an objective response rate of 67% in the HRR‐mutated population, significantly superior to 40% in the control group. In addition, with respect to safety, the most common adverse events were similar across the four agents, mainly anemia, nausea, and fatigue (Table 2). According to an analysis of spontaneous reports submitted to FAERS, Olaparib shows a higher incidence of rare and severe hematologic toxicities, with a prominently elevated risk of myelodysplastic syndrome (ROR = 35.47); Niraparib exhibits distinctive cardiotoxicity (hypertension ROR = 5.49) and a tendency toward neurotoxicity, and Niraparib and Rucaparib have higher incidences of photosensitivity reactions, whereas Talazoparib has the fewest gastrointestinal adverse events [10]. Overall, the clinical benefit of PARP inhibitors is highly dependent on biomarker‐based selection such as BRCA, and the efficacy and safety profiles of different agents require individualized assessment [9].

**TABLE 1:** Summary of efficacy results.

PARP inhibitors	Approved indications (mCRPC)	Pivotal clinical trial	Clinical trial phase	Treatment regimen	Biomarker status	rPFS HR (95% CI)	Median rPFS (experimental group vs. control group)	OS HR (95% CI)	Median OS (experimental group vs. control group)	ORR (experimental group vs. control group)
Olaparib	Monotherapy in mCRPC patients with germline or somatic alterations in 14 HRR genes (based on PROfound)	PROfound [NCT02987543]	Phase III	Monotherapy	BRCA1/2, ATM mutation	0.34 (0.25–0.47; *p* < 0.0001)	7.4 vs. 3.6 months	0.64 (0.43–0.97; *p* = 0.02)	18.5 vs. 15.1 months	33% vs. 2% (95% CI: 4.18–379.18; *p* < 0.001; Cohort A)
Olaparib	Combination with abiraterone in BRCAm patients	PROpel [NCT03732820]	Phase III	Combination with abiraterone plus prednisone	All‐comer	0.66 (0.54–0.81; *p* < 0.001)	24.8 vs. 16.6 months	0.81 (0.67–1.00; *p* = 0.054)	42.1 vs. 34.7 months	Not reported in detail
Rucaparib	in patients with BRCA mutations	TRITON2 [NCT02952534]	Phase II	Monotherapy	BRCA1/2 mutation	HR not provided	Single‐arm study: 10.7 months (95% CI: 8.7–13.2)	HR not provided	Single‐arm study: 17.2 months (95% CI: 14.8–20.0)	45.7% (95% CI: 34.6–57.1; BRCA cohort)
Rucaparib	in BRCAm patients	TRITON3 [NCT02975934]	Phase III	Monotherapy	BRCA1/2 mutations	0.50 (0.36–0.69; *p* < 0.001)	11.2 vs. 6.4 months	0.81 (0.58–1.12; *p* = 0.21)	24.3 vs. 20.8 months	45.1% vs. 17.1%
Niraparib	Combination with abiraterone in BRCAm patients	GALAHAD [NCT02854436]	Phase II	Monotherapy	BRCA1/2 mutations	HR not provided	Single‐arm study: 8.08 months (95% CI: 5.55–8.38)	HR not provided	Single‐arm study: 13.01 months (95% CI: 11.04–14.29)	34.2% (95% CI: 23.7–46.0; measurable BRCA cohort)
Niraparib	Combination with abiraterone in BRCAm patients	MAGNITUDE [NCT03748641]	Phase III	Combination with abiraterone plus prednisone	HRR‐mutated subgroup, BRCA1/2‐mutated subgroup	0.73 (0.56–0.96; *p* = 0.022), 0.53 (0.26–0.79; *p* = 0.001)	16.5 vs. 13.7 months (HRR‐mutated), 16.6 vs. 10.9 months (BRCA1/2‐mutated subgroup)	Data not yet mature	Data not yet mature	Not reported separately (but higher ORR in combination arm)
Talazoparib	Combination with enzalutamide in HRRm patients	TALAPRO‐2 [NCT03395197]	Phase III	Combination with enzalutamide	HRR mutation	0.63 (0.51–0.78; *p* < 0.0001)	NR vs. 21.9 months	Data not yet mature	Data not yet mature	67% vs. 40% (*p* = 0.002)

Abbreviations: ATM, ataxia‐telangiectasia mutated gene; BRCA, breast cancer gene; CI, confidence interval; HR, hazard ratio; HRR, homologous recombination repair; NR, not reported; ORR, objective response rate; OS, overall survival; rPFS, radiographic progression‐free survival.

**TABLE 2:** Summary of safety outcomes.

PARP inhibitors	Common adverse events	Serious adverse events (grade ≥ 3)
Olaparib	Anemia (42.97%), nausea (42.58%), decreased appetite (30.47%), fatigue (26.56%), diarrhea (21.09%)	Anemia (23%), pneumonia (2%), venous thromboembolic events (8%)
Rucaparib	Fatigue (61%), nausea (50%), anemia or decreased hemoglobin (47%), decreased appetite (36%)	Anemia or decreased hemoglobin (24%), neutropenia (7%), fatigue (7%), pulmonary embolism (3%)
Niraparib	Nausea (58%), anemia (54%), vomiting (38%)	Anemia (33%), thrombocytopenia (16%), neutropenia (10%)
Talazoparib	Anemia (49.0%), decreased appetite (20.4%), nausea (20.1%), vomiting (7.3%)	Anemia (46.7%), neutropenia (18.3%), thrombocytopenia (7.3%)

## 4 Discussion

This study systematically analyzes the current status of clinical research on PARP inhibitors in the treatment of prostate cancer, with several key findings: First, the research and development process shows clear phase‐specific characteristics, with Phase II clinical trials accounting for the highest proportion (42.2%), and the number of such trials (*n* = 46) far surpassing other phases. This indicates that most research on PARP inhibitors for prostate cancer has entered the critical mid‐phase efficacy validation stage. Second, drug development is highly concentrated, with Olaparib being the most widely and extensively studied drug, spanning all development phases, particularly Phase II and III, highlighting its core position in this treatment field. Finally, 39.4% of the studies are multinational, multicenter trials, with the United States leading 72 trials, reflecting the internationalization trend in research and development.

Currently, several PARP inhibitors have been approved for the treatment of mCRPC. For example, in 2020, olaparib (OLA) and rucaparib (RUC) were both approved by the FDA for the treatment of mCRPC patients with HRR gene mutations [11]. However, there are differences in the applicability of different PARP inhibitors. Olaparib shows activity in 14 types of DNA repair gene mutations, with the EMA approving its use for patients with BRCA mutations. Additionally, the combination of olaparib and abiraterone has been approved for use in unselected mCRPC patients in Europe, while in the United States, it is approved for mCRPC patients with BRCA mutations [12]; rucaparib, on the other hand, is indicated for DDR gene‐mutated patients who have progressed after treatment with enzalutamide or abiraterone [13]. The next‐generation PARP inhibitor niraparib is currently undergoing clinical trials, with the Phase III AMPLITUDE study evaluating the efficacy of niraparib in combination with abiraterone acetate and prednisone (AAP) in biomarker‐selected metastatic castration‐sensitive prostate cancer (mCSPC) patients. Additionally, based on the MAGNITUDE trial, the combination of niraparib and AAP has been approved for BRCA1/2 mutation patients in the United States, Canada, and Europe [14]. The combination of talazoparib and enzalutamide has been approved in Europe for unselected mCRPC patients and in the United States for HRR‐mutated mCRPC patients, further expanding treatment options [15, 16].

The clinical trials reported in this study have already shown some promising results in their respective regions. This suggests that the survival outcomes for these patients are likely to improve significantly in the next 10–15 years, and the treatment landscape for prostate cancer will undergo new changes. Additionally, training for local physicians on the use of PARP inhibitors in the treatment of prostate cancer should be prioritized.

Future research needs to advance the optimized application of PARP inhibitors in prostate cancer treatment from multiple perspectives. Firstly, most trials indicate that different genetic mutations may lead to varying sensitivities to PARP inhibitors. Therefore, there is a need to deepen the understanding of HRR gene testing and further develop biomarkers to aid in optimizing treatment selection [17]. Secondly, optimizing treatment sequences or drug combinations to delay resistance is an important area of exploration. For example, a Phase II study suggested that sequential treatment with abiraterone followed by enzalutamide resulted in a longer PSA progression‐free interval, delaying the onset of resistance [18]. In addition, the side effects of PARP inhibitors, such as myelosuppression, diarrhea, and fatigue, also require attention. Next‐generation PARP inhibitors, such as AZD5305 and senaparib, have shown increased selectivity and decreased toxicity in preclinical studies, suggesting their potential for improving the safety profile of treatment [19]. In particular, the selective targeting of PARP1 by AZD5305 may reduce the hematologic toxicity seen with nonselective PARP inhibitors while retaining anticancer efficacy [20]. The collaborative advancement of these research directions will collectively promote the precise application of PARP inhibitors in prostate cancer treatment and enhance clinical efficacy.

In conclusion, PARP inhibitors have brought breakthrough progress to the treatment of prostate cancer, particularly metastatic castration‐resistant prostate cancer (mCRPC). However, their widespread application still requires further research to address issues such as resistance, toxicity, and patient stratification. With the development of precision medicine, PARP inhibitors are expected to become an important component of comprehensive treatment for prostate cancer.

## Author Contributions

Y.C. contributed to conception, the design of the study, analysis and interpretation of data, and manuscript drafting; J.C. and R.H. contributed to conception, the design of the study, analysis and interpretation of data; J.L. and J.G. contributed to the design of the study, analysis and interpretation of data, and manuscript drafting; Z.L., T.Y., J.X. contributed to project administration; supervision; funding acquisition, and review. All authors contributed to the critically revised manuscript and provided approval for submission.

## Ethics Statement

This study is a secondary analysis, and we only included publicly available clinical trials.

## Consent

Therefore, it does not involve any violation of patient privacy and does not require additional ethics committee approval or signed informed consent.

## Conflicts of Interest

The authors declare no conflicts of interest.

## Data Availability

The number of all the trials included in the analysis is provided in the appendix. If further assistance is needed, you can contact the rosin@ai‐research.group.
